# The characteristics of depressive symptoms in medical students during medical education and training: a cross-sectional study

**DOI:** 10.1186/1472-6920-8-60

**Published:** 2008-12-11

**Authors:** Sergio Baldassin, Tânia Correa de Toledo Ferraz Alves, Arthur Guerra de Andrade, Luiz Antonio Nogueira Martins

**Affiliations:** 1 Psychiatry and Medical Psychology Disciplines, ABC Regional Medical School, Santo André, Brazil; 2 Psychiatry Department of the Medical Faculty, University of São Paulo, São Paulo, Brazil; 3 Psychiatry and Medical Psychology Department, Federal University of São Paulo, São Paulo, Brazil

## Abstract

**Background:**

Medical education and training can contribute to the development of depressive symptoms that might lead to possible academic and professional consequences. We aimed to investigate the characteristics of depressive symptoms among 481 medical students (79.8% of the total who matriculated).

**Methods:**

The Beck Depression Inventory (BDI) and cluster analyses were used in order to better describe the characteristics of depressive symptoms. Medical education and training in Brazil is divided into basic (1^st ^and 2^nd ^years), intermediate (3^rd ^and 4^th ^years), and internship (5^th ^and 6^th ^years) periods. The study organized each item from the BDI into the following three clusters: affective, cognitive, and somatic. Statistical analyses were performed using analysis of variance (ANOVA) with *post-hoc *Tukey corrected for multiple comparisons.

**Results:**

There were 184 (38.2%) students with depressive symptoms (BDI > 9). The internship period resulted in the highest BDI scores in comparison to both the basic (p < .001) and intermediate (p < .001) periods. Affective, cognitive, and somatic clusters were significantly higher in the internship period. An exploratory analysis of possible risk factors showed that females (p = .020) not having a parent who practiced medicine (p = .016), and the internship period (p = .001) were factors for the development of depressive symptoms.

**Conclusion:**

There is a high prevalence towards depressive symptoms among medical students, particularly females, in the internship level, mainly involving the somatic and affective clusters, and not having a parent who practiced medicine. The active assessment of these students in evaluating their depressive symptoms is important in order to prevent the development of co-morbidities and suicide risk.

## Background

Depressive symptoms are highly prevalent among medical students. Several studies have revealed that medical students are susceptible to high rates of morbidity during their undergraduate years [1-5] and this can be related to impairment in the development of professional, academic, and social skills [6-9]. In addition, this co-morbidity is associated with an increased risk of suicide, evaluated by attempted and completed suicides [10,11].

Helmers *et al*. (1997) compared the presence of depressive and anxiety symptoms among medical students and other disciplines in higher education [12]. The authors found that medical students experienced less stress than law students, graduate students, and the general population, although medical students had elevated scores on stress and depressed mood inventories at the transition from basic-to-clinical training. However, it does not seem to be an adequate comparison, considering that they are different populations, with very different curriculum characteristics and methods of teaching-learning. More recently, Dyrbye *et al*. [13] systematically reviewed the literature reporting on depression, anxiety, and burnout among U.S. and Canadian medical students. The authors concluded that medical school is a time of significant psychological distress for physicians-in-training; however, the current available data was insufficient to draw firm conclusions on the causes and consequences of student distress.

Medical education and training can directly contribute to the development of depression [13] and behavioral problems, such as alcohol and drug abuse [14,15]. During the first semester, there are significant changes in the student's daily habits [16,17]. Other issues may lead to the development of depressive symptoms among medical students, such as work overload, competitive environment, constant pressure of examination/assessment, as well as the vicissitudes of the coursework, which exposes students to several sources of distress from the admission process to graduation, including dealing with traumatic events, such as death and dying, ethical dilemmas, dissecting cadavers, pathologic processes, the first physical examination on a patient [18], the fear of acquiring diseases, feelings of inadequacy, medical hierarchies, and bullying and harassment [19-21]. On the other hand, some authors have focused their studies in identifying risk factors for development of depression in medical students. Some known risk factors for developing affective disorders are gender, lack of family support [22,23], personal history of depressive disorders [24], personal beliefs towards the medical professional [25,26], and the number of years of schooling prior to entry into medical school [27].

The medical education accounts for diversity across different countries; some programs are 4-year graduate entry programs and others are 5 or 6 years undergraduate programs. Many programs have early clinical experience and the boundaries between clinical and preclinical are not exactly clear. In Brazil, we have a 6-year program, and the medical curriculum is divided into basic (1^st ^and 2^nd ^years), intermediate (3^rd ^and 4^th ^years), and internship (5^th ^and 6^th ^years) periods. The curriculum for the basic phase focuses on preclinical disciplines involving studying both structure and function of the cell and the human body (such as anatomy and biochemistry). The intermediate phase focuses on internal medicine disciplines and preliminary clinical experience, with a compilation of courses in the main areas (general medicine, public health, surgery, pediatrics, and gynecology). The internship period is developed in the general hospital and emergency units in a 2-year direct supervision learning action program.

Even though there is an abundance of literature regarding the presence of both depression and depressive symptoms among medical students, it can be difficult to distinguish from the effects of the stress inherent in student life [28]. There is scattered information regarding the specific aspects of these symptoms, such as cognition, somatic, and affective dimensions. None of the above-mentioned studies examined the specific pattern of depressive symptoms during medical education and training. Better knowledge and understanding of the symptoms involved in the depression in medical students could assist in the development of specific target programs (like mentoring and tutoring), thus helping professors and medical educators to better understand and identify students at risk in each year of education or level of training and to reduce the impact of any disturbances in attitudes and behavior which are imperative in order to make the students aware of their own risk factors [29,30]. Therefore, the current study was aimed at evaluating the specific characteristics of the depressive symptoms in medical students, as well as to perform an exploratory analysis to survey possible correlations with several risk factors.

## Methods

The medical school's Ethics Committee approved the study, and written consent forms were obtained from all participating students.

Students were potentially eligible if currently matriculated at the ABC Regional Medical School, a private medical school supported by three cities (ABC) near the São Paulo State Capital. A total of 603 students were found to be potentially eligible. The questionnaires were anonymous and were distributed to all students who were present at the classrooms before their academic activities. Any student who did not fill out or return the questionnaire was considered a "loss." There was an absence of 122 (20.2%) students. There were no refusals to participate in the study. The comparison between absentees and respondents regarding gender and age were not statistically significant (p = .192). The remaining 481 (79.8%) students (21. 9 ± 2.4 years old) from the basic (n = 163, 33.8%), intermediate (n = 164, 34.1%), and internship periods (n = 154, 32.1%) were assessed using the Beck Depression Inventory (BDI) [31], and a questionnaire evaluating personal data (gender, age, having one first degree relative working as a physician, years of study between high school and entering medical school [27], and living alone or with family).

The presence of depressive symptoms among medical students was assessed using the BDI, a 21-item self-report inventory designed to measure the severity of depressive symptomatology. For the BDI, the answers were dichotomized between the presence and absence of major depressive symptoms. The cut-off points for the BDI were as follows: minimal or none (0–9), mild (10–16), moderate (17–29), and severe (30–63). The Portuguese version of the BDI was validated by Gorenstein and Andrade [31] and a more detailed characteristic of BDI psychometrics have been given elsewhere. The internal consistency of the Portuguese version of the BDI is in agreement with the literature (0.81 for non-depressed subjects and 0.88 for depressed patients) [31].

Clinical studies indicate that the many different symptoms of depression can be grouped into a limited number of clusters [32]. Such cluster analysis can also be helpful in describing the particularities of depressive symptoms in a more differentiated manner than the total BDI score alone would permit. Studying such symptom clusters rather than individual symptoms has the advantage of minimizing the number of correlations examined and consequently the risk of committing a Type I error. Therefore the study organized each item from the BDI into three different clusters in order to perform an exploratory analysis of the particularities of depressive symptoms during the medical course of study. In our analysis, the empirical construction of subscales of items from the pool of the 21-item BDI was given preference over a rational approach based on the individual items' face validity, in order to avoid "inductive bias" caused by the analysts' perceptions of the cognitive field. In order to avoid the intrinsic disadvantages of factor analysis outlined above, the factors were derived by using cluster-analytical techniques. Unlike factor analysis, cluster analysis groups the elements of the analysis (i.e., items and persons) into an appropriate number of mutually exclusive subsets, so that a more straightforward interpretation can be achieved when the analysis is successful. The first factor, affective cluster (the sum of scores on items 1, 4, 10, 11, and 12 from the BDI), represents the core symptoms of a depressive mood, based on the following symptoms: 1, sadness; 4, dissatisfaction; 10, episode of crying; 11, irritability; and 12, social withdrawal. The second factor, cognitive cluster (items 2, 3, 5, 6, 7, 8, 9, 13, 14, and 20), assesses the following cognitive aspects: 2, pessimism; 3, sense of failure; 5, guilt; 6, expectation of punishment; 7, dislike of self; 8, self-accusation; 9, suicidal ideations; 13, indecisiveness; 14, change in body image; and 20, somatic preoccupation. The third factor, somatic cluster (items 15, 16, 17, 18, 19, and 21), assesses the presence of the following characteristics: 15, slowness; 16, insomnia; 17, fatigue; 18, appetite; 19, loss of weight; and 21, loss of sexual interest. In addition, cluster scores were computed as the means across all items assigned to a particular cluster (the mean was given preference over the sum of the individual item scores to account for differences in the number of items per cluster). The possible score for each of these clusters were as follows: affective cluster, a range of 0–21 and a maximum possible score of 30; cognitive cluster, a range of 0–28 and a maximum possible score of 60; and somatic cluster, a range of 0–14 and a maximal possible score of 36.

### Statistical analysis

Statistical analyses were performed using the Window's Statistical Package for Social Sciences (version 13.0). Between-group comparisons of depressive symptoms in students from three medical period were divided in basic (1^st ^and 2^nd ^years), intermediate (3^rd ^and 4^th ^years), and internship (5^th ^and 6^th ^years) using variance analysis (ANOVA) with *post-hoc *Tukey corrected for multiple comparisons after the Kolmogorov-Smirnov test (normality) were carried out. The investigation of significant depressive cluster symptoms in each year was separately carried out using variance analysis (ANOVA) with *post-hoc *Tukey. This was an exploratory investigation aimed at ascertaining whether or not depressive symptoms might be related to a determined period of the medical course of study in order to generate hypotheses for future studies. Pearson correlations between depressive scores were performed upon the entire medical student cohort and within the three periods. Finally, in order to verify which demographic and risk or protective factor variables most significantly influenced the BDI scores in the overall medical student sample, we conducted a multiple-regression analysis using the BDI total score as dependent variable was conducted, involving gender, age, course periods, years to get into medical school, first degree parent as a physician, living alone or with a family as independent factors ("enter method"), and a logistic regression for BDI > 16 using the cut-off to create a pragmatic clinical parameter based in the those used during a Portuguese validation: BDI > 16 to detect a disphoric range and BDI > 20 to detect a considered a depression range [31]. Using this cut of we have obtained a ROC curve with a sensitivity of 75% and specificity at 53%. The significance level used was p < .05.

## Results

### Study Sample

Of the 481 students participating in the study, 40.5% were males, 90.6% lived with family, 17.1% had at least a two-year gap between the end of high school and entering medical school, and 28.2% had a parent as a physician. Table 1 summarizes the socio-demographic characteristics of the sample.

**Table 1:** Demographic characteristics of medical students

Periods	Basic	Intermediate	Internship	Total	p
	
Sample n (% of regular matriculated)	163 (79.1%)	164 (83.7%)	154 (76.7%)	481 (79.8%)	
Age – m (sd)	20.0 (2.0)	21.9 (1.5)	24.0 (1.9)	21.9 (2.4)	<.001^a^
Male n (%)	56 (34.8%)	72 (44.3%)	62 (41.1%)	190 (40.5%)	.242^b^
Female n (%)	102 (30.2%)	92 (55.7%)	85 (58.9%)	279 (59.5%)	
Living with the family n (%)	155 (97.1%)	140 (86.0%)	131 (88.8%)	426 (90.6%)	.011^b^
Having a parent as MD n (%)	44 (28.2%)	56 (33.8%)	33 (22.9%)	133 (28.2%)	.113^b^
> 2 years to enter medical school n (%)	31 (20.3%)	28 (16.9%)	21 (14.7%)	80 (17.1%)	.516^b^
Mean years of studying to enter medical school – m (sd)	1.7 (1.0)	1.7 (0.8)	1.6 (0.9)	1.7 (0.9)	.337^a^

Statistical analyses performed: ^a ^ANOVA; ^b ^Chi-square

In the basic period, there were 163 students with the following characteristics: the mean age was 20.0 years (range, 17–31 years); 34.8% were males; 97.1% lived with family; 20.3% had at least a two-year gap between the end of high school and entering into medical school; and 28.2% had a parent as a physician. In the intermediate period, there were 164 students with the following characteristics: the mean age was 21.9 years (range, 20–35 years); 44.3% were males; 86.0% lived with family; 16.9% had at least a two-year gap between the end of high school and entering into medical school (n = 28); and 33.8% had a parent as a physician. Finally, in the internship period, there were 154 students with the following characteristics: the mean age was 24.0 years (range, 21–36 years); 41.1% were males; 88.8% lived with family; 14.7% had at least a two-year gap between the end of high school and entering into medical school; and 22.9% had a parent as a physician.

### Investigation of depressive symptoms among the entire medical student population

The general mean of the BDI for the medical course of study was 9.1 ± 7.6. Considering the BDI cut-off points, 184 (38.2%) were depressed (mild depression, n = 120 [24.9%]; moderate depression, n = 53 [11%]; and severe depression n = 11 [2.3%]). With regard to the characteristics of the depressive symptoms presented by the entire medical student population, the cluster analysis showed the following mean score ± S.D., range, and maximum possible score for each of these clusters: affective cluster, mean ± S.D. = 2.4 ± 2.5, range 0–21, maximum possible score = 30; cognitive cluster, mean ± S.D. = 3.9 ± 3.8, range 0–28, maximum possible score = 60; and somatic cluster, mean ± S.D. = 2.9 ± 2.4, range 0–14, and maximum possible score = 36. There was a significant difference between BDI total scores and gender distribution: female (n = 279, BDI, 9.8 ± 8.1) and male (n = 190, BDI, 8.1 ± 6.8; t-test, T = 2.363, p = .019).

### Cluster analysis of depressive symptoms in separate years among medical students

Table 2 shows the mean BDI scores in the medical course of study grouped according to the period of the course. The mean BDI scores for each period were as follows: basic (8.6 ± 7.9); intermediate (7.0 ± 6.9); and internship period (11.7 ± 7.2). The ANOVA showed a significant association between the year level and the mean BDI scores (F = 9.282, degrees of freedom = 5, p < .001). *Post hoc *Tukey for each period showed that there were significant differences between the internship period in comparison to both basic and intermediate periods.

**Table 2:** Analysis by clusters analysis of depressive symptoms assessed by Beck Depression Inventory scores among medical students (n = 481).

Periods	Basic	Intermediate	internship	Total	ANOVA
Cluster	m (sd)	m (sd)	m (sd)	m (sd)	
Affective	2.1 (2.7)	1.8 (2.0)	3.5 (2.4)^a,b^	2.4 (2.5)	<.001
Cognitive	4.0 (3.9)	3.2 (3.4)^c^	4.7 (3.6)	3.9 (3.8)	<.01
Somatic	2.8 (2.2)^c^	2.1 (2.2)^d^	3.8 (2.6)^b^	2.9 (2.4)	<.001

BDI total	8.6 (7.9)^c^	7.0 (6.9)	11.7 (7.2)^b^	9.1 (7.6)	<.001

significant post hoc Tukey comparing each year of medical course

^a ^p < .001 in comparison to basic period medical school

^b ^p < .001 in comparison to intermediate period medical school

^c ^p < .01 in comparison to internship period medical school

^d ^p < .05 in comparison to basic period medical school

In the ANOVA investigating cluster analysis among the three periods (basic, intermediate, and internship), significant differences with regard to the affective cluster (2.1 ± 2.7, 1.8 ± 2.0, and 3.5 ± 2.4, respectively, F = 22.220, p < .001), cognitive cluster (3.9 ± 3.9, 3.1 ± 3.5, and 4.7 ± 3.8, respectively, F = 6.493, p = .002), and somatic cluster (2.8 ± 2.2, 2.1 ± 2.2, and 3.8 ± 2.6 respectively, F = 19.927, p < .001) were observed. *Post hoc *Tukey for each period showed that there were significant differences between basic and intermediate periods with the internship periods in the affective cluster (p < .001); between the intermediate period with the internship period in the cognitive cluster (p < .01); and between the basic period and the internship period (p < .01) and between the intermediate period and the internship period (p < .001) in the somatic cluster.

### Exploratory preliminary investigation of possible protective/risk factors for depression among medical students

A multiple regression analysis was conducted on the BDI scores in order to ascertain the effect of other demographic variables in the development of depressive symptoms in medical students. Linear regression for a total BDI revealed that there was a significantly higher risk for development of depressive symptoms in course periods (CI 95%, .873:2.580, p < .001). Others significant risk factors assessed by linear regression in the sample were female gender (CI 95%, -3.140:-.353, p < .05) and those not having a parent who was a physician (CI 95%, -3.395:-.311, p < .05). Significant demographic characteristics assessed by logistic regression for BDI > 16 in the sample were course period (CI95%, 1.284:2.592; p < 0.01) and those not having a parent who was a physician (CI95%, .168:.805, p < .05) There were no other significant findings, including living alone (without family) (CI 95%, .567:5.013, p = .348) and having at least a two-year gap between the end of high school and entering into medical school (CI 95%, .814:.3.059; p = .177).

## Discussion and conclusion

This is the first study that directly evaluated, in a cross-sectional design, the characteristics of depressive symptoms by applying clusters. Higher total BDI scores during the internship period (11. 7 ± 7. 2) were observed. On the other hand, the total BDI scores were lower in the intermediate period (7.0 ± 6.9). The means of somatic, cognitive, and affective clusters decreased during this part of the course 1.5-fold and tiredness decreased by one-half. These increased rates of BDI scores during the internship [33,34] period of medical school are associated with a decrease in student health, and probably refers to periods when professors and educators should be aware about suicidal thoughts and risk. Future studies specifically addressing this aspect should be designed in order to investigate the suicide risk among medical students.

The interpretation of both BDI score as means and standard deviation, and a second analysis using a cut-off for depression in BDI > 16 was in agreement with the proposition of the experience of depression as a continuum, and that sub-threshold or sub-syndromal depression differs quantitatively rather than qualitatively from major depression [35]. The hypothesis was that the nature of the depressive experience would differ in intensity (quantitatively), but not in kind (qualitatively), in individuals with mild versus severe depression. In short, the cluster analysis was used to empirically sort the symptom profiles of the analogue participants of this study. The analysis by clusters showed that the principal cluster responsible for BDI scores was the affective cluster, which includes a core evaluation of mood disorders, namely sadness, lack of pleasure, crying, irritability, and losing interest in people. This aspect was significantly different among the medical education and training periods, with higher scores in the internship period, followed by the basic course and lower scores in the intermediate course. The cognitive cluster, which included aspects of hopelessness, failure, criticism, self-punishment, self-blame, indecision, guilt, self-appearance, and somatic worrying was also higher in the internship period, probably would be associated to negative feelings, such as fear and insecurity [36], related to entry into the internship period. Frequently pre-internship students experience the fear of "knowing nothing" [37], and are insecure about the physical examination of other human beings [38,39]. Finally, the somatic cluster refers to insomnia [40], tiredness, loss of appetite [41], loss of weight and sexual interest, therefore it is not surprising that in internship period the scores are significantly higher reflecting the nights on call, without sleeping, devoid of friends and family support, facing the death of patients, with reduced time of pleasure activities [42-44], and fear regarding the future and residency program examination [45].

The relevance of analyzing the characteristics of depressive symptoms among medical students might help educators in dealing with different patterns of depression and developing specific target strategies [46]. The higher affective cluster of depressive scores in the internship may affect since the beginning the feelings of pleasure and compassion of the physician-patient relationship [47,48]. The higher cognitive cluster during the internship occurs at the same time of deceptions with the institution and health system, fears of the professional future, failures, performance, even dislike own appearance, this in general not good, due the lack of sleep and leisure and fewer physical and sexual activities. Taken all together, it might suggest that the possible development of coping mechanisms during medical education and training is responsible for lower scores in the intermediate period. However, these mechanisms are not necessarily functional [49,50], healthy or effectives, perhaps influencing the future professional [51]. Coping strategies developed in response to a stressful event can be subdivided into "functional" (problem solving, cognitive re-structuring, and seeking social and emotional support) or "dysfunctional" (problem avoidance, social and emotional isolation, and guilt and self-criticism) [52].

The study also showed that female students were more susceptible towards developing depression symptoms than male students. Gender comparisons in the academic profession, prior to, during [39] and after the medical course of study sho ws that the gap between males and females in medicine is getting narrower. Even though academically there is no significant gender differences, several non-cognitive aspects often shows a different pattern among males and females. It is interesting to note that Hojat et al. (1999) studying a large sample of medical students, indeed observed that male and female medical students have a different pattern of stress response [53]. Further investigation on gender difference among medical students is required in order to develop a specific-gender program to prevent stress-related mental disorders. In the exploratory analysis of protective and risk factors for developing depressive symptoms, it was found that studying more than 2 years to get in medical school was a risk factor for depression. In Brazil, there is a very special condition the "vestibular" [27] examination has to be passed after high school in order to enter any Brazilian medical school. Even though the medical profession is highly distressful, there are many students competing for each medical school vacancy. Of interest to note that having a parent as a physician seems to be a protective factor. It may occur that being a son/daughter of a physician is probably associated to lower idealism concerning the medical course of study [50] and the consequent development of social or affective coping skills [54]. Further studies that specifically address this aspect should be conducted in order to investigate which particular aspects of familiar support are possibly protecting the students. Depressive symptoms, off course, are not prevalent only in the medical students of this study, and as said Hickie and Davenport, it is necessary understanding depression as a major condition in different cultural settings [55].

The findings of this study must be interpreted with caution due to its methodologic shortcomings, including the use of self-administered inventories and no structured interviews for clinic diagnoses. However, it is unlikely that our results of higher scores in the internship period were limited only to the presence of non-specific depressive symptoms due to the BDI that has been often been used to separate depression\non-depression by applying a cut-off at 17 points {Department of Psychiatry, 1998 #7901}, therefore, the total of 13.3% with moderate/severe depressive symptoms (BDI, 17–63) is still concerning. Even though the inclusion of an exploratory analysis of the different subscales of BDI could be considered a little forced, this was done in order to generate future hypotheses to identify specific depressive symptoms in the different periods of medical education and training, once previous studies have assessed either the presence of depressive symptoms comparing only the total BDI scores. Better knowledge regarding specific depressive symptoms could assist medical educators in the development of suitable strategies to deal with the particularities of depression in medical students.

We should consider if the cohort characteristic of each year or period might have influenced our results. We observed that the 2^nd ^year students seems to be quite different from the others years, in term of the lowest response rate (64%) and gender. This might have influenced the differences observed in the score. The absence of 20% of the medical students in class might limit the generalization of our finding, However, it is feasible to think that absence might be also be related to the presence of depressive disorder, particularly in the 2^nd ^year. Several studies have related the presence of depressive symptoms and absence at school or work [57]. The current study with 80% of all students of a medical school is based on self-answer questionnaires that might be associated to a bias of minimizing or maximizing symptoms. However, it has the advantage of maintaining anonymities and allowed for the study of a large sample of medical students. Finally, even considering that medical students possibly have more depression/anxiety symptoms than other students [58], it is necessary to consider aspects related to medical education and training, such as an important risk factor to the development, sustaining and/or worsening of emotional distress. However, our cross sectional design prevented us from this particular analysis.

Finally, an additional limitation to this study is its cross-sectional design, where the different subgroups may differ in respects not directly attributable to the stage of education. We have not measured intra-individual symptoms changes during medical education and training using a repetitive measure and a longitudinal design. Therefore, the presence of individual variability can not be excluded and, given the cross-sectional nature of the study, in which it was not possible to define the temporal relationship between cause and consequence, we cannot state that the associations observed in the study are of a causal nature However, our approach allowed us to assess the presence of depressive symptoms in a large sample including up to 80% of all medical students regularly matriculated at our medical school. Despite these limitations, this study indeed contributes to the literature on medical education.

In conclusion, our findings of a greater frequency and severity higher depressive scores among medical students on internship period years and lower scores on intermediate period, as well the better knowledge of the characteristics of each cluster symptoms, are involved could help us to develop programs (like mentoring and tutoring) specifically designed for each level to prevent the development of depression and its consequence, as well as, preparing our medical students and faculty members for their daily practice and teaching perceptions [59].

## Competing interests

The authors declare that they have no competing interests.

## Authors' contributions

Each author have made substantial contributions to conception, design, acquisition, analysis and interpretation of data, have been involved in drafting the manuscript, revising it critically for important intellectual content and given final approval of the version to be published.

## About the authors

SB, MD, MsC, is a Professor of Psychiatry and Medical Psychology Disciplines, Medical School, ABC Foundation, where he also coordinates the counseling and medical student service.

TCdTFA, MD, PhD is a Professor of Psychiatry and Medical Psychology Disciplines, Medical School, ABC Foundation, where she coordinates the Affective Disorders Ambulatory and is also a researcher of the Psychiatry Department of the Medical Faculty, University of São Paulo.

AGdA, MD, PhD is Chairman Professor of the Psychiatry and Medical Psychology Disciplines, Medical School, ABC Foundation, and Professor of the Psychiatry Department of the Medical Faculty, University of São Paulo.

LAN-M, MD, PhD is a Chairman Professor of Psychiatry Department at Federal University of São Paulo, where he is also a coordinator of the Medical Service Ambulatory for Residents.

## Pre-publication history

The pre-publication history for this paper can be accessed here:


